# Matrix metalloproteinases and their tissue inhibitors after selective laser trabeculoplasty in pseudoexfoliative secondary glaucoma

**DOI:** 10.1186/1471-2415-8-20

**Published:** 2008-10-21

**Authors:** Mauro Cellini, Pietro Leonetti, Ernesto Strobbe, Emilio C Campos

**Affiliations:** 1 Department of Surgery Science and Anesthesiology, Ophthalmology Service, University of Bologna, Italy

## Abstract

**Background:**

The aim of this study was to assess changes in metalloproteinases (MMP-2) and tissue inhibitor of metalloproteinases (TIMP-2) following selective laser trabeculoplasty (SLT) in patients with pseudoexfoliative glaucoma (PEXG).

**Methods:**

We enrolled 15 patients with PEXG and cataracts (PEXG-C group) and good intraocular pressure (IOP) controlled with β-blockers and dorzolamide eye drops who were treated by cataract phacoemulsification and 15 patients with pseudoexfoliative glaucoma (PEXG-SLT group). The PEXG-SLT patients underwent a trabeculectomy for uncontrolled IOP in the eye that showed increased IOP despite the maximum drug treatment with β-blockers and dorzolamide eye drops and after ineffective selective laser trabeculoplasty (SLT). The control group consisted of 15 subjects with cataracts. Aqueous humor was aspirated during surgery from patients with PEXG-C, PEXG-SLT and from matched control patients with cataracts during cataract surgery or trabeculectomy. The concentrations of MMP-2 and TIMP-2 in the aqueous humor were assessed with commercially available ELISA kits.

**Results:**

In PEXG-SLT group in the first 10 days after SLT treatment a significant reduction in IOP was observed: 25.8 ± 1.9 vs 18.1.0 ± 1.4 mm/Hg (p < 0.001), but after a mean time of 31.5 ± 7.6 days IOP increased and returned to pretreatment levels: 25.4 ± 1.6 mm/Hg (p < 0.591). Therefore a trabeculectomy was considered necessary.

The MMP-2 in PEXG-C was 57.77 ± 9.25 μg/ml and in PEXG-SLT was 58.52 ± 9.66 μg/ml (p < 0.066). TIMP-2 was 105.19 ± 28.53 μg/ml in PEXG-C and 105.96 ± 27.65 μg/ml in PEXG-SLT (p < 0.202). The MMP-2/TIMP-2 ratio in the normal subjects was 1.11 ± 0.44. This ratio increase to 1.88 ± 0.65 in PEXG-C (p < 0.001) and to 1.87 ± 0.64 in PEXG-SLT (p < 0.001). There was no statistically significant difference between the PEXG-C and PEXG-SLT ratios (p < 0.671).

**Conclusion:**

This case series suggest that IOP elevation after SLT can be a serious adverse event in some PEXG patients. The IOP increase in these cases would be correlated to the failure to decrease the TIMP-2/MMP-2 ratio.

**Trial registration:**

Current Controlled Trials **ISRCTN79745214**

## Background

Argon laser trabeculoplasty has become the standard method of treatment for medically uncontrolled open angle glaucoma [1,2]. It has been in use since 1979 when it was first described by Wise [3] and different types of lasers with various wavelengths have been investigated. The first laser used was an argon laser, but recently a Q switched frequency doubled Nd:YAG laser was proposed for use in trabeculoplasty [4,5], described as selective laser trabeculoplasty (SLT).

A number of theories have been proposed to explain the effect of argon laser trabeculoplasty (ALT) and SLT on aqueous outflow. The mechanical theory suggests that ALT causes photocoagulative damage to the trabecular meshwork (TM), which results in collagen shrinkage and subsequent scarring of the TM [6]. The cellular theory is based on the migration of macrophages, due to coagulative necrosis induced by laser burns, which phagocytose debris and clear the TM [6]. SLT selectively targets pigmented TM cells, while it spares collateral cells and tissue from thermal damage and can maintain the architecture of the TM [7]. Experiments have shown that there is also a third mechanism involved in laser trabeculoplasty which causes an increase in the production of metalloproteinases (MMPs) induced by the TM photocoagulation [8,9]. These enzymes are responsible for the extracellular matrix (ECM) turnover. Their action is countered by a family of tissue inhibitors, the tissue inhibitors of metalloproteinases (TIMPs).

The aim of this study was to assess changes in the MMP-2 and TIMP-2 values in a group of patients with pseudoexfoliative syndrome glaucoma (PEXG) in whom SLT failed to decrease intraocular pressure (IOP).

## Methods

We assess the MMP-2 and TIMP-2 values in the aqueous samples of 15 patients with pseudoexfoliative glaucoma (PEXG-SLT group) (8 males and 7 females aged from 58 to 66, mean 63.8 yrs) that presented post-SLT IOP elevations. As control groups we enrolled 15 patients with pseudoexfoliative glaucoma and cataracts (PEXG-C group) (9 females and 6 males, aged from 61 to 68, mean 65.4 yrs) and 15 subjects with cataracts (4 males and 11 females aged between 62 and 74 years, mean 68.3 yrs), All patients were recruited from the Glaucoma Disease Service and from the Ophthalmology Services of the S. Orsola-Malpighi Hospital, Bologna.

Patients with other ocular or systemic disorders, such as inflammatory diseases or diabetes mellitus, were excluded from the study.

The study was approved by the institutional ethic committee of the S. Orsola-Malpighi hospital (ref: 2007-007744-98). Before enrolment, patients were informed of the procedures and the aim of the study following which they signed a written consent form.

After a complete ocular examination, including assessment of visual acuity, anterior segment biomicroscopy, applanation tonometry and visual field evaluation (Humphrey 30.2 full threshold programme), the PEXG-C patients who had good intraocular pressure (IOP) control with β-blockers and dorzolamide eye drops underwent cataract phacoemulsification. The PEXG-SLT patients underwent a trabeculectomy for uncontrolled IOP in the eye that showed increased IOP, despite being under maximum drug treatment with β-blockers and dorzolamide eye drops and after having received selective laser trabeculoplasty (SLT).

SLT was performed with a Selecta 7000 (Q switched, frequency doubled, 532 Nd:YAG laser) using 50 non-overlapping applications in the inferior 180° of the trabecular meshwork, with a spot size of 400 μm and pulse duration of 3 ns. The initial energy used was 0.8 mJ. The energy was increased or decreased until bubble formation and then was decreased by 0.1 mJ for the remainder of the treatment.

The laser treatment was considered to be effective when a decrease of 20% in the IOP was obtained as compared to the base values or when the visual field was stabilized.

In the first 10 days after SLT treatment a significant reduction in IOP was observed: 25.8 ± 1.9 vs 18.1.0 ± 1.4 mm/Hg (p < 0.001), but after a mean time of 31.5 ± 7.6 days IOP increased and returned to pretreatment levels: 25.4 ± 1.6 mm/Hg (p < 0.591). Therefore a trabeculectomy was considered necessary.

Before enrolment, all patients were informed of the procedures and the aim of the study and they signed a written consent form.

Aqueous humor was aspirated during surgery from patients with PEXG-C, PEXG-SLT and from matched control patients with cataracts. Thus 80–100 μl of aqueous humor was aspirated through an ab externo limbal paracentesis site using a tuberculin syringe with a 30-gauge needle. Meticulous care was taken to avoid touching intraocular tissues and to prevent contamination of the aqueous samples with blood. The samples were immediately frozen in liquid nitrogen and stored at -80°C.

The concentrations of MMP-2 and TIMP-2 in aqueous humor were assessed by ELISA using commercially available enzyme immunoassay kits (Biotrak; Amersham Biosciences, Piscataway, NJ) according to the manufacturer's instructions.

Differences in MMP-2 and TIMP-2 levels between groups, as measured by ELISA in aqueous humor samples, were statistically evaluated using the Wilcoxon signed rank test and a p < 0.05 was considered significant. Data are presented as means ± standard deviation (SD). The analysis was performed using SSI (version 11, Systat Software Inc., San Jose, California, USA) for Macintosh.

## Results

There was a significant difference between controls and PEXG-C in terms of MMP-2 (p < 0.013) and TIMP-2 (p < 0.005) values. The same statistical significance was found in PEXG-SLT both for MMP-2 (p < 0.013) and TIMP-2 (p < 0.005).

The TIMP-2/MMP-2 ratio in controls was 1.11 ± 0.44. This ratio increased to 1.88 ± 0.65 in PEXG-C (p < 0.001) and to 1.87 ± 0.64 in PEXG-SLT (p < 0.001).

The PEXG-C and PEXG-SLT ratios were not significantly different (p < 0.671).

The results are summarised in Tables 1 and 2.

**Table 1:** Levels of MMP-2 and TIMP-2 in PEXG with cataract, PEXG after SLT and Cataract groups.

	Cataract	PEXG-C	p < 0.05	PEXG-SLT	p < 0.05
MMP-2 ng/mL	55.04 ± 10.24	57.77 ± 9.25	0.013	58.52 ± 9.66	0.013
TIMP-2 ng/mL	54.43 ± 8.18	105.19 ± 28.53	0.005	105.96 ± 27.65	0.005
**TIMP-2/MMP-2**	1.11 ± 0.44	1.88 ± 0.65	0.001	1.87 ± 0.64	0.001

Levels of MMP-2 and TIMP-2 in aqueous humor and TIMP-2/MMP-2 ratio of patients with PEXG and cataract (PEXG-C), PEXG after selective laser trabeculoplasty (PEXG-SLT) and in healthy patients with cataract.

**Table 2:** MMP-2 and TIMP-2 levels in PEXG with cataract and PEXG after SLT

	PEXG-C	PEXG-SLT	p < 0.05
MMP-2 ng/mL	57.77 ± 9.25	58.52 ± 9.66	0.066
TIMP-2 ng/mL	105.19 ± 28.53	105.96 ± 27.65	0.202
**TIMP-2/MMP-2**	1.88 ± 0.65	1.87 ± 0.64	0.671

Levels of MMP-2 and TIMP-2 and their ratio in patients with PEXG with cataract (PEXG-C) and in patients with PEXG after selective laser trabeculoplasty (PEXG-SLT)

## Discussion

Our study shows that the TIMP-2/MMP-2 ratio is higher in patients with PEXG-C and PEXG-SLT than in the cataract control group.

After the initial success of selective laser trabeculoplasty treatment with a reduction in IOP of 20% compared with the baseline values, eyes with PEXG-SLT showed a progressive increase in IOP, which, after a mean of one month returned to similar values to those presented before treatment. The TIMP-2/MMP-2 ratio in the PEXG-SLT patients did not differ significantly from that in PEXG-C patients (p < 0.671).

SLT has been shown to be effective in long- and short-term treatment of POAG and is comparable to ALT [5,9] but some studies have reported post-operative IOP increase as a complication of SLT [5,9-11]. The mean incidence of increased IOP after SLT is 2.4% but no author has specified whether eyes in which SLT had failed had special trabecular meshwork features. Only Harasymowycz et al. reported the complication of IOP elevations after SLT in patients with a heavily pigmented trabecular meshwork [11] that required surgical trabeculectomy. They suggested that increased IOP may be related to trabeculitis or increased angle scarring [11].

The active mechanism of selective laser trabeculoplasty is still the subject of debate, although it has been suggested that besides the action focused on the pigmented cells of the trabecular meshwork [4] there is also an enzymatic action that stimulates the production of metalloproteinases [8,9], as already demonstrated experimentally with other types of laser [13].

Metalloproteinases (MMPs) are a very numerous and heterogeneous family of zinc-dependent proteinases that fulfil the role of degrading the ECM components to maintain homeostasis [14,15]. The various enzymes are distinguished on the basis of the type of media that they degrade: collagenases, gelatinases, stromelinases and the membrane-type MMPs [17-19].

MMPs are secreted in the form of zymogens (pro-enzymes) that are activated by a selective proteolysis by part of a serine proteinase. Once activated, MMPs begin their proteolytic action on the ECM [17-19]. To guarantee the balance between ECM degradation and deposition there are tissue inhibitors of metalloproteinases (TIMPs) that block them [13,15,18,19].

MMP-2 is the major metalloproteinase secreted after laser therapy and is inhibited by TIMP-2 [8,20,21]. In pseudoexfoliative glaucoma (PEXG), the enzyme balance between MMP-2 and TIMP-2, already impaired by the pseudoexfoliative syndrome (PEX), is seriously altered even compared with POAG [22,23].

PEX is a degenerative fibrillopathy characterised by the production and accumulation of extracellular fibrillar material, not just in the anterior segment of the eye [24], but also in numerous extraocular tissues [25,26]. At an ocular level, there is atrophy of the iris dilator muscle fiber cells [27,28], degeneration of the irideal pigment with dispersion of melanin, peripupillar atrophy and an increase in the trabecular meshwork pigmentation [29-31]. Furthermore, at the iris level there is also a general vasculopathy with deposits of pseudoexfoliative material that lead to a thinning of the basal membrane and degeneration of the adventitial cells [32,33], which explains the hypoperfusion and neovascularization of the iris [34,35].

As for the trabecular meshwork, a typical feature is the excessive accumulation of the extracellular matrix material in the juxtacanalicular tissue [36]. This accumulation explains the increased resistance to aqueous outflow found in eyes with PEX [37,38]. The deposits that are found in the iris vessels and the trabecular meshwork in PEX consist of numerous substances, including laminin, fibronectin, alpha-elastin, tropoelastin, fibrillin and P-amyloid protein [24].

P-amyloid protein plays an important role in the alteration of ECM homeostasis since it can increase MMP production and activity [39].

In our study levels of MMP-2, the predominant gelatinase in aqueous humor, and TIMP-2 in PEXG and controls were in line with previous findings [22,23].

Our results suggest that in this case series of PEXG, a particular type of glaucoma in which there is upregulation of MMP-2 production with a marked increase in the relative inhibitors [22,23], the changes in the TIMP-2/MMP-2 ratio that can be obtained with laser trabeculoplasty are insignificant and thus are unable to decrease intraocular pressure.

## Conclusion

In conclusion, we can be said that in some cases of PEXG may be a significant risk of IOP elevation after SLT. The IOP increase in these cases would be correlated to the failure to decrease the TIMP-2/MMP-2 ratio.

Further studies are needed for evaluation of this phenomenon and determination for incidence.

## Competing interests

The authors declare that they have no competing interests.

## Authors' contributions

MC performed the SLT, drafted the manuscript and reviewed the literature. ES recruited the patients from the Glaucoma Disease Service of the S. Orsola-Malpighi Hospital. PL examined the patient in the time and ECC performed both cataract and trabeculectomy surgery and review the manuscript.

## Pre-publication history

The pre-publication history for this paper can be accessed here:


